# The San Bernardino, California, Terror Attack: Two Emergency Departments’ Response

**DOI:** 10.5811/westjem.2016.1.29720

**Published:** 2016-01-12

**Authors:** Carol Lee, Elizabeth Walters, Rodney Borger, Kathleen Clem, Gregory Fenati, Michael Kiemeney, Sakona Seng, Ho-Wang Yuen, Michael Neeki, Dustin Smith

**Affiliations:** * Loma Linda University Medical Center, Department of Emergency Medicine, Loma Linda, California; † Arrowhead Regional Medical Center, Department of Emergency Medicine, Colton, California

## Abstract

On December 2, 2015, a terror attack in the city of San Bernardino, California killed 14 Americans and injured 22 in the deadliest attack on U.S. soil since September 11, 2001. Although emergency personnel and law enforcement officials frequently deal with multi-casualty incidents (MCIs), what occurred that day required an unprecedented response. Most of the severely injured victims were transported to either Loma Linda University Medical Center (LLUMC) or Arrowhead Regional Medical Center (ARMC). These two hospitals operate two designated trauma centers in the region and played crucial roles during the massive response that followed this attack. In an effort to shed a light on our response to others, we provide an account of how these two teaching hospitals prepared for and coordinated the medical care of these victims.

In general, both centers were able to quickly mobilize large number of staff and resources. Prior disaster drills proved to be invaluable. Both centers witnessed excellent teamwork and coordination involving first responders, law enforcement, administration, and medical personnel from multiple specialty services. Those of us working that day felt safe and protected. Although we did identify areas we could have improved upon, including patchy communication and crowd-control, they were minor in nature and did not affect patient care.

MCIs pose major challenges to emergency departments and trauma centers across the country. Responding to such incidents requires an ever-evolving approach as no two incidents will present exactly alike. It is our hope that this article will foster discussion and lead to improvements in management of future MCIs.

## INTRODUCTION

On December 2, 2015, terrorists attacked the Inland Regional Center (IRC) in San Bernardino, California, killing 14 Americans and injuring 22 in the deadliest attack on U.S. soil since September 11, 2001. The city of San Bernardino is situated approximately 60 miles east of Los Angeles and has a population of 215,000 and a median income of $38,385.^1^ Many of its citizens rely on the resources provided by entities such as the IRC, where the shooting occurred. The IRC is a not-for-profit organization that provides services for over 31,000 people with developmental disabilities and their families, including children,^2^ and employs nearly 600 people.

The shootings began at approximately 11:00 AM when two terrorists entered a conference hall at the IRC and started shooting. The initial available information was limited and it was unknown to the hospital providers whether the incident represented workplace violence or a terrorist attack. The mode and appearance of the attack, however, was concerning for terrorism. When patients were being treated and transported to the emergency department (ED) by first responders, the shooters had not been apprehended.

Although emergency personnel are familiar with multi-casualty incidents (MCIs), as well as violent acts, the shootings that occurred that day were unprecedented. Since the attack, reports of the heroism of the victims have been well documented in the lay press. In the coming months our medical community will undoubtedly review our system’s response to the attack in depth, including the key roles our community hospitals provided. We applaud the professionalism and valor of the responders in the field and grieve the loss of those who died on site. We wanted the opportunity to do more. However, in an effort to provide timely information to other emergency physicians who may be preparing for such an event, we are providing an account of how two separate teaching hospital EDs responded to the attack.

### Loma Linda University Medical Center and Children’s Hospital Response

Loma Linda University Medical Center and Children’s Hospital (LLUMC&CH) is a Seventh-day Adventist institution located in Loma Linda, CA, approximately three miles from where the incident occurred. An academic center with 714 beds, it is the only Level I trauma center for adults and children in San Bernardino County and serves the Inland Empire (San Bernardino and Riverside counties). The ED has 38 adult beds, 18 pediatric beds, and four Express Care beds and sees more than 70,000 patients per year.

### Activation

LLUMC ED personnel first became aware of the incident when a firefighter, dispatched to the scene, alerted our Mobile Intensive Care Nurse (MICN) directly through a phone call. No “official” notification through our county “Comm Center” or the emergency medical communications network (ReddiNet) had yet been received at the time of this initial call. The County Comm Center was contacted for verification, but none was yet available. On-scene responders estimated that there were at least 20 severely injured patients.

The charge nurse was notified and nursing administration began the emergency response, activating our disaster plan based on the initial call. We established an incident command center in the nursing administration office, which is located away from the ED. The ED was full at the time of the attack and all admitted patients were quickly sent upstairs as per our normal disaster plan. We relocated the remaining patients away from the six adult resuscitation rooms. We also cleared and readied an additional five beds in the pediatric ED to receive critical patients. The pediatric and adult EDs are separate spaces but physically attached making mutual support fluid. Disaster supply carts were brought to the outside triage area located in the adjacent parking lot, staff were assigned specific duties, and handheld phones were distributed to key personnel. A wireless computer set up outside allowed for order entry to the electronic medical record.

The ED attending physician was also aware and with the anticipated number of victims, she contacted the trauma surgeon on call. Additional trauma surgeons were placed on standby and the trauma medical director contacted the operating room (OR) manager who worked to clear rooms for victims. Five ORs were made immediately available with assurances others would become available in short order. There was no resistance to holding planned elective surgeries and ORs were kept on standby for about four hours. This process allowed our patients immediate OR access once it was determined they needed surgery. ED physician leadership was paged to respond to the ED.

Hospital security established a corridor outside the ED and all vehicular traffic was diverted from the area. Access to the ED, as well as other portals of entry to the hospital, was controlled. A small number of non-shooting related patients and one person looking for a family member involved in the shooting were allowed through the corridor to access the ED after being checked by security. A sizeable number of local law enforcement and fire personnel soon arrived, providing a needed additional security and response capability. They also successfully addressed the bomb threat our hospital received later that afternoon. Media were maintained outside the safety corridor and quickly established themselves on the street side opposite the ED entrance. Approximately four media trucks were visible from the entrance of the ER, although more were likely on campus at different locations.

Our disaster plan includes setting up a triage tent outside the ED, including basic supplies. This was completed in less than 20 minutes from the initial activation. Registration personnel prepared patient labels and charts so that incoming patients could be quickly registered, necessary for placing orders and charting, as well as for tracking. Patients already in the waiting room were moved to any available ED beds out of the resuscitation area as well as into an Express Care associated with the ED, and later into the triage tent.

With the activation of the disaster plan, the Department of Environmental Health and Safety, which oversees disaster planning for the medical center and university, was notified. A representative was sent to the ED command center, along with hospital administration, security, public information, information technology, and communications.

### Response

At the time of the activation, the ED was staffed with two attending physicians on the adult side and one on the pediatrics side. With the paging of the ED physician leadership, four additional attendings arrived to the ED a short time after the activation. Serendipitously, an emergency medicine (EM) resident education conference was in progress on campus at the time of the attack. An attending physician in the lecture hall had been alerted to the shootings by a CNN “breaking news” text alert and two attendings were already making their way to the ED to investigate when official word was received through the disaster communication system. Physicians at conference were notified and prior to the first patient arriving to the ED, 26 EM residents, three pediatric emergency medicine (PEM) fellows, seven EM attendings, and five PEM attendings arrived in the ED to assist with the response.

Two emergency physicians were assigned to the triage area, along with nursing and ancillary staff. As it was known that the IRC provides services for children, there was concern that there might be pediatric victims as well as adults. Five resuscitation beds in the Peds ED were ready for pediatric or adult patients. A total of 11 resuscitation beds were initially available. Each resuscitation bed was manned with an ED attending and senior EM resident prepared to receive a patient. Two procedure nurses, one nurse for documentation, an ED tech, and a respiratory therapist (RT) were also assigned to each resuscitation bed. Additional physicians were placed on standby in an area out of the resuscitation zone.

The chief of trauma surgery also responded to the ED with another three attending surgeons, approximately five trauma residents, and a trauma nurse practitioner. Three additional surgery attendings, who had been called in, also arrived to the ED giving us immediate access to seven attending surgeons. ED trauma care at our facility is collaborative, and the EM and surgery teams integrated into respective roles smoothly. Patients were placed alternately in the adult and pediatric EDs so that resources would be more evenly distributed. The trauma surgeons determined the order in which they would attend the patients. The on-call surgeon was the last to be assigned, allowing him to be available for other patients not involved in the incident. We had initially notified Comm Center that we would be able to take 10 “immediate” patients, and with the response that was available felt we could treat up to 50 additional patients.

Additional nursing personnel quickly arrived in the ED as well. In addition to implementing our official staffing processes for disasters response, many nurses aware of the attack from other sources came to the ED to assist with the care of patients. Approximately 50 nurses and techs were available. Respiratory therapy successfully mobilized extra personnel so that each resuscitation bed was set up with an RT and ventilator. Another RT was available for providing care to the other patients in the ED. Radiology staffed two auxiliary computed tomography (CT) scanners, and x-ray stood ready with the portable machine. The director of the blood bank responded to the ED with our “Code Black” blood products (O negative and positive blood, cryoprecipitate, FFP, platelets) and two “runners” to obtain more blood products as required. We had been informed that our blood supplier was within the incident area and would not be available to us. The blood inventory was assessed and seemed ample, with 60 units of PRBC immediately available. However without knowing how many victims might need transfusion, contingency plans were made. Our affiliated and other local hospitals were contacted, in case additional blood would be necessary, and transport was placed on standby. The directors of the laboratory and pharmacy, along with a pharmacist, were also available in the ED for immediate access to those services. A pharmacist is regularly physically present in our ED and ensured we had what was needed to respond to the patients’ needs.

At the time of the attack approximately 15 patients were in the waiting room. No announcement was initially made to the waiting room regarding the shooting and the waiting patients were moved to other areas. Care to the other patients in the ED was continued by the physician’s assistant and nurse practitioner already working in the ED. ED attendings and residents not assigned to the resuscitation teams also continued to see the other patients. During the response period, multiple other critically ill patients were managed, including a patient with acute stroke, a patient in respiratory failure who required immediate intubation, and a patient with an acute arterial occlusion of the upper extremity. A Level B trauma activation for a motor vehicle accident with multiple serious injuries was already en route to our facility prior to the shooting notification and was successfully stabilized in the ED before admission. We used the time between the arrival of the fourth and fifth shooting victims to see the less acute patients in the triage tent, some of whom were able to be discharged directly from there, albeit without the usual discharge paperwork.

### Patient Timeline

The first patient arrived at 11:44 with an anterior chest gunshot wound. The patient was alert but tachypneic. Bedside ultrasound was negative for a pericardial effusion, but concerning for decreased lung sliding. A chest tube was placed in the ED. Although initially hemodynamically stable, during the ED course the patient became hypotensive, underwent multiple transfusions, and was taken emergently to the OR by the trauma team.

Patient number two presented at 11:48 with a laceration to the chest, as well as multiple wounds to the face, arm, and leg. The patient remained alert in the ED, and was hemodynamically stable. A focused assessment with sonography for trauma (FAST) scan was negative, and multiple plain radiographs of chest and extremities showed no fractures, but multiple metallic fragments. The patient continued to have bleeding from leg wounds, concerning for vessel injury. CT angiography showed a possible venous injury and the patient was taken to the OR for exploration and washout.

The third patient arrived at approximately 11:50 in critical condition with multiple gunshot wounds including wounds to the chest, hypotension, and altered mental status. FAST scan was positive for bilateral pneumothoraces. Multiple metallic fragments and rib fractures were seen on plain radiographs. We placed a chest tube and the patient was transfused packed red cells, but persistent hypotension resulted in the patient going directly to the OR for further evaluation.

The fourth patient arrived five minutes later, approximately 11:55, with multiple gunshot wounds to the pelvis and leg. One of these wounds with copious bleeding was sutured in the ED for hemostasis. Radiographs showed multiple pelvic fractures. FAST was positive for blood in the bladder. The patient was given tranexamic acid and underwent massive transfusion protocol for hypotension and was taken immediately to the operating room.

The fifth patient arrived sometime after the initial influx of patients. Before the patient arrived, it was noted that several news helicopters were overhead with high resolution cameras focused on the ED parking lot, in addition to the press stationed at the street opposite the ED entrance. A line of nursing staff used sheets to create a visual barrier while the patient was transported from the ambulance to the ED. This act of compassion was recognized and appreciated by the patient and family. Fortunately the patient’s injuries were not immediately life threatening and the patient was admitted to the trauma service rather than the OR.

### Discussion of Loma Linda University Medical Center and Children’s Hospital Response

This incident underscored the importance of disaster training. With a disaster drill recently conducted in our hospital, the initial set up of the ED with equipment, communications, triage, and security occurred seamlessly. ED providers were familiar with the process and their duties. Having the infrastructure in place served to organize and focus the response.

Assigning treatment teams (triage, resuscitation, existing ED patients) also worked well. With this “zone” perspective, we were able to provide care to the victims as well as to our current ED patients and keep the department running.

Having blood bank immediately available was invaluable. Three of our patients required multiple transfusions, one of whom received multiple blood products.

The presence of the surgery attendings and immediate access to the ORs was crucial. While the patients received stabilizing measures in the ED, since it was unknown exactly how many patients would be received, ED procedures were minimized and were performed in the OR. This allowed our resuscitation teams to prepare for the arrival of additional patients.

Communication between the key personnel and the hospital incident command worked reasonably well. Access to several handheld phones was key, but having pre-assigned numbers for the various positions, or pocket cards to write in each individual’s extension would have been helpful. A major issue, however, was the communication challenges with the on-site command and first responders. We received limited information regarding patient injuries prior to the patients’ arrival, which made the planning for care more difficult. Also, there was confusion regarding the number of patients we were receiving. Most of the confusion was related to “unofficial” calls from the scene. Believing that three additional patients were on their way, we held surgeons and operating rooms for extra time before learning that no more patients were being transported. It should be noted that the information received from Reddinet was accurate. From a more personal perspective, incident information from the site was limited, and ED staff was receiving multiple messages from friends and family, even from as far away as Afghanistan. Throughout the hospital, employees were trying to get accurate details. Knowledge that the shooters were still at large only added to the concerns. At one point, it was rumored that two shootings that occurred during the same time frame at other local venues were related to the incident at the IRC. So many hospital employees were attempting to live stream newscasts that our IT department recognized it might slow down hospital communication services. For a short period of time, Internet access was restricted to hospital and emergency operations only. Employees were advised to centralize their access to news, or were able to continue the use of their telecommunications networks.

Safety and crowd control was another concern. The ED was inundated with essential and nonessential personnel offering assistance. This could have impacted efficiency and posed a potential security threat. Staging of additional personnel in an area near but outside the ED is a better option. This would provide access to personnel when needed but would also allow verification of each individual and accountability for who is on site. This is especially important should a second simultaneous incident occur. For example, during our response to the multiple shooting victims our institution received a bomb threat. Because explosive devices had been found at the IRC it was felt to be a credible threat. Notification of staff, patients, and families in the hospital was discussed in the incident command, and it was felt that all should be notified. Our ED executive director informed patients in the waiting room and gave them the option of leaving. Only one patient chose to leave. Senior administrative personnel went to each inpatient unit and informed the charge nurse, who was asked to inform staff, patients and family. Physicians received information about the bomb threat through our communication system about 20 minutes after the initial threat was received. Additionally, an email and text/page was sent to all personnel. Knowing who is in the department and who has already left is crucial for both security and accountability.

Similarly, there should be an established location for family assistance. We had family members calling and arriving to the ED trying to locate potential victims. We were able to divert these calls to our social workers, but this was done ad hoc. There is a plan for family assistance in our disaster plan, but because the hospital was so close to the incident site, this portion of the plan and not been initiated prior to their arrival.

This response, while focused in the ED, was successful only because of the willingness of all of our hospital partners to fully participate in necessary activities to decompress the ED, expedite patient flow, and provide the best care possible to all our patients. Collaboration led to a successful response to a heinous attack.

### Arrowhead Regional Medical Center’s Hospital Response

Arrowhead Regional Medical Center (ARMC) is a 456-bed university-affiliated teaching hospital operated by the County of San Bernardino. The hospital is located in Colton, California, which is approximately five miles from the scene of the terrorist attack that occurred on December 2, 2015. It is the only American College of Surgeons (ACS) verified level II trauma center in the region. Additionally, it operates a regional burn center, a primary stroke center, a behavioral health center, and a tertiary referral center providing more than 40 specialty care services. ARMC also supports multiple training programs including a four-year EM residency with 33 residents. ARMC’s ED is one of the busiest in the state, handling more than 116,000 patient visits each year.

### Activation

On December 2, 2015, ARMC was notified that we had an active shooter scenario in the nearby city of San Bernardino. This notification came to us directly from the city of Colton police department to the ED charge nurse on duty around 11:10 AM, even before the incident was officially posted on the regional emergency broadcast system called ReddiNet at 11:17 AM. Reports were that this was a multi-casualty incident and we were to expect around 12 gunshot wound (GSW) victims. Three ED attending physicians were already on duty that morning; one of them is additionally trained as a tactical medicine SWAT team member. Because we were able to immediately mobilize additional ED attending physicians and residents who were already on the hospital campus attending a weekly lecture, he decided to respond to the scene of shooting along with SWAT team members.

### Response

Immediately, we fully staffed our eight trauma beds. Each bed had anesthesia, EM, and trauma surgery personnel. In addition, we converted four of our medical beds into lower acuity trauma beds, bringing our total to 12 available trauma beds. We had three trauma nurses in house that morning, but three additional nurses responded to the call for extra help. The charge nurse also sent five ED nurses into the trauma resuscitation area. An ED tech was placed at each bed.

All together, we had available five EM attending physicians, 20 EM residents, and several physician assistants (PA-C) in the ED. This workforce was divided into receiving and assisting with newly arriving shooting victims and continuing care of existing ED patients. In addition, we had four attending trauma surgeons and eight general surgery residents respond. Overall, we had enough staff to assign at least one attending (either trauma surgeon or EM attending) and two residents to each trauma bay. We had four attending anesthesiologists present, enough to assign two trauma bays to each anesthesiologist. Hospital administration including the medical director and the chief of surgery also responded to the ED.

We assigned two nurses to each trauma bay, preferably using the combination of one trauma nurse paired with an ED nurse. We also notified the RTs to prepare all available vents to be mobilized (we had the ability to place over 30 victims on vents if the need occurred) along with preparation for additional intubation trays and supplies. Additional RTs also responded so that one RT could be assigned to each trauma team.

Beyond our ED, eight operating rooms were placed on standby. All elective non-emergent surgeries were held. Two CT scanners were made available for immediate use and all but emergent CT studies were put on hold. Three X-ray techs were placed in the hallways outside the trauma bays for portable studies. Furthermore, the blood bank, sterile processing, laboratory and pharmacy were also put on alert.

In addition to mobilizing staff and resources in preparation, we asked for assistance from several inpatient services and bed control in order to free up as many ED beds as possible. Particularly, the internal medicine service and pediatric service responded quickly by completing admitting processes for several patients pending admission in the ED. Bed control and hospital administration assigned inpatient beds quickly and facilitated these patients’ movement out of the ED even before the first victim arrived. All patients already in the waiting room or in the ED rooms were seen and evaluated in a usual manner by a separate ED crew. No one was sent home without proper medical evaluation. All discharged patients were provided with usual instructions and follow up. However, during our lockdown period, efforts were made to redirect new, stable patients to other hospitals after confirming that they had the capacity and the capability to treat them. No outside tent was used as we were able to clear a large number of ED beds quickly.

Many ED staff not on duty voluntarily called the charge nurse or the charge physician and offered to report. In addition, many on campus but not necessarily on duty either physically reported to the ED or called in, offering assistance in any way possible. In total, we had more than 70 additional staff members from various services physically show up to the ED. Most of them were re-directed to a separate area where they were asked to wait for further instructions. Because our hospital runs disaster drills regularly and we had just completed one within the past month, most of the staff members in the hospital were familiar with their roles and the processes involved in a large-scale disaster.

During the event, we were notified that there might have been shootings at the nearby Patton State Hospital. This report came in directly from one of the SWAT members working in the first shooting scene via a text message to the ED physician in charge. Patton State Hospital is a forensic psychiatric hospital located within the County of San Bernardino. It operates 1,527 beds and typically houses those incompetent to stand trial or found not guilty of a crime by reason of insanity. This immediately raised our concern that possibly a coordinated attack was being carried out on multiple psychiatric/medical/public health facilities operated by government entities. We also received reports that the shooters might have been San Bernardino county employees. At this point, ARMC went on lockdown; no persons were allowed in or out. SWAT members deployed from multiple surrounding cities took posts outside the hospital with snipers on the rooftops. Armed police officers from multiple cities and precincts (including those Colton city police officers already stationed at ARMC) took posts within the hospital. Each ambulance approaching the ED was stopped by law enforcement at the road block outside of the hospital, and occupants in each ambulance were checked by SWAT before being allowed to pull up to the ambulance bay. We were asked to be aware of any persons in the area not wearing hospital badges. At times, none of us knew for sure the identities of the patients we were receiving, but we were assured by the law enforcement that all our patients were pre-screened at the scene before being loaded onto the ambulances.

### Patient Timeline

At the end of the event, 14 shooting victims were pronounced dead at the scene and were not transported. We at ARMC received six injured patients: five were transported via EMS and one was driven in by a police officer. In total, 21 patients were transported via EMS to local hospitals: five to ARMC, five to LLUMC, two to Community hospital of San Bernardino, two to Kaiser Hospital Fontana, two to Kaiser Hospital Ontario, two were flown to Riverside County Medical Center, two to San Antonio Community Hospital, and one to St. Bernadine’s Medical Center. In all, 22 patients were evaluated and treated, and all survived their injuries. Of the six patients that were brought to ARMC, one went directly to the OR, one was discharged home from the ED, and the rest were admitted to either ICU’s or trauma floor units with various injuries. Overall, there were no inpatient fatalities.

### Discussion of Arrowhead Regional Medical Center’s Response

A week after the event, a debriefing was held; participants included hospital administration, trauma surgeons, anesthesiology and ED attending physicians, along with ED charge and trauma nurses. Overall consensus was that our response was well-organized, well-run, and well-staffed. We were incredibly proud of the teamwork that was displayed and amazed by everyone’s willingness to step up and help out in such a challenging situation. However, we identified several issues regarding security/safety, communication, and crowd control that we thought could be improved upon.

First, there was a concern that when everyone in the trauma bay was fully gowned and masked, there was no way to identify the person’s role. There should be additional tags or banners with labels (such as “team captain,” “airway doctor,” “trauma nurses,” etc.) to help identify each team member. Anyone without a proper label should be questioned, as there was always a concern of breach of security, where a shooter might unknowingly be allowed access into the ED and cause more casualties or that a shooter could actually be one of the patients. Related to this topic of security, we discussed whether all patients should have been completely undressed at the ambulance bay to check for any hidden weapons or explosive devices before they were allowed inside of the hospital. For this particular incident, every patient was searched beforehand by the law enforcement officials. However, for future scenarios, this should not be assumed and we should consider a more standard way for law enforcement, paramedics, and receiving hospitals to communicate that patients have already been searched and cleared as a potential threat.

Communication was identified as another area that needed improvement. Initial information we received was limited and sometimes inaccurate. We could not verify the exact number of patients we would be receiving, nor the severity of their injuries. Each patient en route was called into the base station, but the information we received was patchy in terms of their acuity, stability, and types of injuries. Additionally, the first notification came directly from the field and not through the regional emergency communication network, making it difficult for us to confirm its legitimacy.

Finally, crowd control was a major challenge. We estimated that close to 70 people were in the ED/trauma area at one time or another, and it became difficult to identify who was essential and who was not. Non-essential staff members were directed to wait in the cafeteria located one floor below, but more and more people continued to present themselves to the ED throughout the day and in some way hindered security and efficient operation.

## CONCLUSION

MCIs are unique events that bring forth a bevy of challenges to EDs and trauma centers across the country. They test the technical abilities of providers, stretch the resources of multiple hospitals, and rely heavily upon the communication skills at each level of patient care. Responding to such incidents requires an ever-evolving approach, as no two incidents will ever present exactly alike.

While MCIs traditionally are taught and practiced through scenarios involving natural disasters or accidental trauma, it is undeniable that we are currently in an era where it is crucial to prepare ourselves for MCIs of a different nature, namely the active shooter.

In 2015 alone the U.S. experienced the following mass shootings: nine deaths at Emanuel African Methodist Episcopal Church in Charleston South Carolina on June 18; five deaths at a Navy support center in Chattanooga, Tennessee on July 16; nine deaths at Umpqua Community College in Roseburg, Oregon, on October 1; three deaths at a Planned Parenthood clinic in Colorado Springs, Colorado, on November 29; 14 deaths in San Bernardino on December 2.

It is our hope that this article will foster discussion that leads to improvement in our management of MCIs while shedding light on what it was like to manage a live incident while dealing with the possibility of an on-site hospital threat.
